# Customer Behavior on Purchasing Channels of Sustainable Customized Garment With Perceived Value and Product Involvement

**DOI:** 10.3389/fpsyg.2020.588512

**Published:** 2020-12-21

**Authors:** Zhenfang Li, Jia Yuan, Bisheng Du, Junhao Hu, Wenwen Yuan, Lorenzo Palladini, Bing Yu, Yan Zhou

**Affiliations:** ^1^School of Business, Ningbo University, Ningbo, China; ^2^Center for Collaborative Innovation on Port Trading Cooperation and Development, Ningbo University, Ningbo, China; ^3^Department of Management, Faculty of Law, Economics and Finance, Luxembourg University, Luxembourg City, Luxembourg

**Keywords:** purchasing, customer to manufactory, C2M, customized garment, product involvement, perceived value

## Abstract

Online shopping for customized garments has become the fastest-growing field of the Chinese eBusiness market. Most consumers not only limit themselves to buying standardized garments but also want to buy garments customized to their preferences. This phenomenon has pushed the fashion textile and apparel industry to change its supply chain operations to meet the customization demand. Besides, the fashion textile and apparel industry also want to study how different channel factors will affect consumers' perceived value and further influence consumers' purchasing decisions. We initiated this study and empirically tested more than 200 experienced consumers. This study collaborated with a fashion textile and apparel company that aims to implement customized product lines soon. Based on the perceived value theory and risk management theory, we investigated whether product involvement and channel identification on supply chain design will affects potential customized product consumers' purchasing decisions. The findings reveal that channel recognition affects consumer decisions by having a positive impact on their perceived value. The perceived risk and shopping channel involvement of consumers have a negative impact on their perceived values and channel selections. In addition, product involvement has a moderating effect on the relationship between channel's perceived risk, perceived values, and channel selections as well.

## 1. Introduction

The rapid evolution of development plans like Industry 4.0 and Made in China 2025, as well as emerging information technologies, such as Cloud Computing, Big Data, and the Internet of Things, plays an important role in the development of garment production (Damodaram and Ravindranath, 2010; Ngai et al., 2014; Dong et al., 2020; Gu et al., 2020). Many garment firms in the apparel industry have, in fact, introduced high-end technology to design innovative, stylish, and customized products to attract more potential customers. Because of the intense market competition, which is one of the main characteristics of this industry, firms involved design and produce lots of garments, which results in an abatement of their own products lifetime (Mostard et al., 2011). The final result of this cause and effect chain is that the leftover garment inventory becomes an important restrictive factor to the development of brand clothing enterprises, especially the ones located in China.

In order to deal with this issue, apparel firms decide to use all the channels they know, from online channels to offline ones, from their own domestic market to other international markets, to sell their garments and provide services for their consumers. However, the recent trend, which sees consumers pursuing their own personalities and paying more attention to wearing both customized and high-quality clothes, is posing a real threat to these firms (Ladhari et al., 2019; Zhang and Zheng, 2020). Moreover, the environmental pollution issue, generated by the garments' manufacturing process, is now more than ever occupying a great deal of capital in the fashion textile and apparel industry, and furthermore, it brings attention to the operation management process and all the related research fields (Shen, 2014; Turker and Altuntas, 2014; Jia et al., 2015). The mismatch between the consumer's growing demand for personalized products and the supply side's increasing leftover inventory attracts many new businesses into this apparel industry (Bharadwaj et al., 2009; Hsu et al., 2014). We also know that along with the customizing factor increase, there are more challenges in the manufacturing process.

Going back to some history, we find out that the development of production models underwent three main eras: mass production, mass customization, and now, personalized customization. These three main eras have, in turn, generated three representative models, which are respectively, the MTS (Make to stock), the MTO (Make to Order), and the C2M (Customer to Manufactory). The last one especially, the C2M (or C2B), is recognized as the best sustainable business model (Zhang et al., 2015; Zhou et al., 2016) because one of its essential features, which perfectly suits the apparel industry in this fast-growing Big Data era, is garment customization. The recent development on customized business models, especially C2B, can be found in the state-of-the-art review article (Zhang et al., 2019).

Garment customization is an important business model and strategy from which consumers can get personalized products and the firm can get, in return, higher customer satisfaction and less accumulated inventory; moreover, it represents an effective way to increase the cash flow turnover and decrease the responsiveness time (Alptekinoğlu and Corbett, 2008; Dong et al., 2012; Choi et al., 2013; Nayak et al., 2015; Tookanlou and Wong, 2020). The C2M model has proven to be able to enhance a firm to better understand its customer needs, and, more than that, it meets and equals both supply and demand targets at the first design and manufacturing stage. Based on the above description, it is easy to deduce that apparel industry's members are eager to understand how to optimize their supply chain operations and build suitable and effective channels to attract those potential consumers that prefer to buy customized products.

Mass customization is not a brand-new topic; it has been under discussion for several years (Zipkin, 2001), but there have always been a number of obstacles for its implementation. For example, Lampel and Mintzberg (1996), in their studies, analyzed and summarized the apparel customized industry and divided it into five different strategies: pure standardization, segmented standardization, customized standardization, tailored customization, and pure customization. Another important issue to point out concerns the communication channels between customers and suppliers. Several channels can be built up to accept consumers' orders: an official website and a portable smart device app are maybe the most popular channels for consumers to use. The E-Business Research Center of China (Editing Team, 2019) released an annual report showing that in 2016 about 67% of the consumers placed their orders from a smartphone app, 10% of them used an official website, and 23% of the consumers went through other channels.

Personalized customization is a modern production mode able to combine the advantages of both personalized and customized production, which poses customer focus as its key issue by focusing on producing the right products according to the customers' individual tastes and target prices. In today's apparel industry, this phenomenon has been translated into a detailed and meticulous process in which garments are manipulated from their standard versions following the shortest and most cost-effective way. Especially in the Chinese developing market, this production mode has become a key marketing strategy. The Chinese market is characterized by an enormous demand, for which a personalized production process is necessary. On the other hand, as the Chinese citizens' average income has been exponentially increasing, customers' desires have changed and shifted from standard products to more personalized and fashionable design, which requires a drastic change in the production model (Shao, 2020).

Although this transition does not come without any difficulties, it brings with it a number of advantages; top of the list is the decreased level of fierce competition. Thanks to the expanded degree of difference in the possible choices given to the consumers, the chance of a shift from a producer A to another producer B is decreased, which means the market is much more stable and delineated. Moreover, using the personalized customization production mode, suppliers are able to guide customers demand by giving them a range of choices for their customized product, which will optimize the supply chain efficiency, enlarge their market share, and strengthen their competitive advantage (Tookanlou and Wong, 2020; Zhang and Zheng, 2020).

Nowadays, China, from the point of view of the C2M development, which innovative business models have recognized as the fourth technology revolution after steam engine, electrification, and automation, is the most advanced country. A C2M model is based on the close interaction between consumers and businesses, most often trough mobile phone apps, from which businesses acquire knowledge on the consumers' target price, fashion desires, tastes, and many other data that enable companies to better serve and market their products; consumers in turn have the possibility to purchase exactly what they want for exactly the price they are willing to pay, and they also get direct payment methods and flexibility in terms of the available products. But the C2M business model does not refer only to this. It implies a much wider range of innovation in different processes, such as automation, intellectualization, networking, customization, and energy saving, which are essential in the conception of a sustainable customized garment supply chain. Consumers' channel selection for customized products has great effects on the supply chain's performance. Thanks to this new developing trend of customized products, manufacturers can make attractive layouts for their channels and thus gain a competitive advantage in this specific market. Obviously, one of the necessary conditions for this process to take place is that the product manufacturer and the consumers dispose of some channels to communicate their preferences and, sometimes, their limits. Nowadays, this is not just more than possible, it is actually routine thanks to the increasing popularity of smartphones (Editing Team, 2019), wearable devices and other intelligent terminal devices, which give the customized garment manufacturer easy access to customers' information, preferences, and data, and allow the producer to get in contact with the customer even during the manufacturing and distribution processes [here, it is important to mention the newly available technologies like the 3D printing machine (Zhou et al., 2016), artificial intelligence (Gu et al., 2020), and big data (Dong et al., 2020)].

The remainder of this paper is organized as follows. In section 2 we review the relevant research theoretical background and hypotheses. In section 3 we describe the methodology we use and the research design. Conclusions, implications, and further research are given in section 4.

## 2. Theoretical Background and Hypotheses

### 2.1. Perceived Channel Value

The customer's perceived value is a widely used concept in a number of disciplines. Zeithaml's study (Zeithaml, 1988) on perceived value has led to the development of an independent theoretic study, which sees it as a completely independent field of research. He pointed out that perceived value is the result of both the consumer's ability to consider products based on the overall perception of gains and losses and the evaluation of the product's utility. Since Zeithaml's research, many outstanding scholars have expanded the scope of this theory into serval aspects. In general, most of these research's results show that the consumer's perceived value is subjective, multidimensional, hierarchical, comparative, and contingent. Moreover, many researchers have done lots of in-depth excavation analysis on the argument from the above-mentioned dimensions' points of view. For instance, Park et al. (1986) divided the perceived value into three parts: functional value, symbolic value, and experiential value. Based on this study, Sweeney and Soutar (2001) proposed a Perl model consisting of four dimensions: functional value price, functional value quality, emotional value, and social value.

Parasuraman and Grewal (2000), on the other hand, hold different viewpoints; they argued, in fact, that the concept of this dynamic should be composed of acquisition value, transaction value, usage value, and redemption value. Another key topic to consider here concerns the connection between consumer's purchase intention and purchase decision. It is well-known that the consumer's purchase intention results in the final purchase decision (Peña-García et al., 2020). However, the purchase intention is aroused by a series of consumer's psychological activities, which are the result of each customer's ability to measure the pros and cons of products, and their related behaviors: we are speaking about customers' perceived value. As a kind of subjective evaluation metric, customer's perceived value is used to measure consumers' profits and losses given by the actual purchase of a specific product, and it is a helpful tool in the analysis and research of the purchase intention and purchase decision relationship. Tam (2004), for example, found that when consumers choose any kind of online shopping channel, when it comes to making a purchase decision, the perceived value carries a much higher weight than the consumer satisfaction. Chu et al.'s conclusions, based on Tam's work, show that utilitarian value, hedonic value, and perceived risk have a direct impact on consumers' repetition of purchasing decisions, and the relationship between perceived value and purchase intention is usually positively correlated (Chiu et al., 2014). In a number of multi-channel environments and markets, the perceived values for customers on the same product are totally different according to the channel used. These differences may have a huge impact on the consumer's choice of the final purchase channel. For example, in fact, some scholars, such as Kwon and Jain (2009), pointed out that the perceived value of practical and hedonic values have significant impacts on consumer channel selection. Furthermore, many other researchers have discovered that the perceived value has a positive effect on the consumer's willingness to change their purchase channel (Ström et al., 2014).

Furthermore, Zheng et al. (2017) extend their research to investigate how the relationships among customers will affect their perceived value propositions on social commerce platforms. Their findings reveal that on social commerce platforms, user relationships can affect participators through information quality and information credibility. Gan and Wang (2017) also studied the effects of perceived benefits on purchase intention in a social commerce context with utilitarian, hedonic, and social values. Meanwhile, Jiang et al. (2018) studied the effects of perceived value from consumers' experience in emerging markets with different retailer brands. In fact, the perceived value of the consumer, at the time of purchase, comes from two aspects: the purchase of the product and the situation at the time of purchase; in fact, in a multi-channel environment, customer's perceived value researchers should analyze in-depth both product value and channel value as the main factors. Based on the above analysis, the following hypothesis 1 is proposed.

**Hypothesis 1**. *Consumers perceived value for online shopping channels has a positive impact on the choice of online shopping channels*.

### 2.2. Perceive Channel Risk

The term risk first appeared in the economics and decision science fields. It is generally believed that it refers to the probability of negative events' occurrence. The risk can be divided into two categories: subjective risk and objective risk. In 1960, Raymond Bauer's perceived risk theory (Bauer, 1960), extending the concept from the psychology subject to the consumer behavior studies, clearly pointed out that the consumer's unpleasant uncertainty in shopping would make the consumer perceive the risk, and that same perceived risk is based on the consumer's subjective cognitive risk. After that, Cunningham (1967) argued that perceived risk is a combination of two factors, uncertainty, and consequences, which, in turn, are based on the consumers' subjective judgment. The perceived risk, therefore, is both inconsistent with the objective risk and influenced by each consumer's own risk attitude. Masoud (2013) believed that online shopping perceived risk is the result of the consumers' subjective judgments on the possibility and severity of adverse outcomes.

The uncertainty of perceived risk leads to the uncertainty of the consumer's final decision; in order to measure the risk of consumer perception and its impact, therefore, Dowling (1986) analyzed them from different dimensions to investigate the usage of the perceived risk concept in consumer research. This work is well-known and cited in quite some literatures. For instance, based on it, Yi et al. (2020) studied the effects of perceived risk in the tourism industry of the sharing economy, and they then analyzed a case study of Airbnb to explain the effects. Forsythe and Shi (2003) extended the research into the online shopping era, investigating both the consumer patronage and risk perceptions. The consumer's perceived risk affects the online shopping behavior, which can eventually explain why the development of online shopping is hampered: it does not only provide products faster and easier but even bears some part of the consumers' risk, a kind of risk that is present in any kind of transaction and that can particularly affect the consumers' decisions when it comes to choosing their shopping channels. Hur and Cassidy (2019) study the perceptions and attitudes toward sustainable fashion design, which reveals that there are internal (personal and organizational levels) and external (social and cultural levels) challenges to incorporating sustainability into the fashion design process. Hur (2020) extended the perceived risks and consumption values into the area of secondhand clothing, which leads to environmentally friendly and sustainable consumption. They found five main values identified among secondhand clothing consumers: economic, self-expressive, hedonic, environmental, and social contribution values. Perceived value has the most direct impact on consumer's shopping decisions. In addition, as the other important factor, perceived risk not only affects the consumers' purchase intention but also has a negative impact on the perceived value. We therefore propose the following hypotheses: 2 and 3.

**Hypothesis 2**. *Consumer's perceived risk for online shopping channels has a negative impact on perceived value*.

**Hypothesis 3**. *Consumer's perceived risk for online shopping channels has a negative impact on consumer choice*.

### 2.3. Consumer Risk Attitude

Risk attitudes refer to the extent of risk that consumers are willing to accept under uncertain conditions. Different consumers have different risk attitudes due to their habits, environment, and culture; these can directly affect their response to the uncertainty and ultimately affect their decision making. In general, speaking about the classification of risk attitudes, the definition given by Nobel Prize Laureate Kenneth Arrow is commonly used. It divides risk attitudes into three types, namely, risk-seeking, risk neutrality, and risk aversion (Arrow, 1965). In contrast to the risk-neutral consumers, the risk-seeking consumers prefer to participate thrill-seeking and high-risk activities, while the risk-averse consumers prefer to participate in activities with fewer risks. Therefore, consumer's risk attitude affects the perceived risk level. Many research findings show that consumers with different risk attitudes have different risk perceptions in the same channel. Comparing with the risk-averse consumers, the risk-seeking consumers perceive less risk. The study conducted by Alhakami and Slovic (1994) confirmed that there is a negative relationship between consumers with risk attitude and perceived risk. Pavlou (2003) found that different risk attitudes will also have significant impacts on consumers' shopping behavior. Suki and Suki (2017) investigate the consumers' attitudes toward online group buying and find that website trustworthiness was the strongest predictor of consumers' attitude, which leads to the implications that online retail managers should improve their transaction security mechanisms and Internet technology to dwindle consumers' perceived risks in terms of financial, product, and time risks. Wang et al. (2018) also study those phenomenon for U.K. consumers with a moderated mediation model. Based on the above analysis, we propose the following hypothesis 4.

**Hypothesis 4**. *Consumer's risk attitude has a regulatory effect on perceived risk and channel selection*.

### 2.4. Product Involvement

The term involvement was first derived from self-involvement by Sherif and Cantril (1947), where it refers to the individual's involvement level. Krugman (1965) introduced this theory into the marketing research and in other applications extensively. Most of the academic scholars believed that the involvement is an important measurement to evaluate consumers' personal feelings in all kinds of matters. Usually, consumer involvement is divided with respect to their characteristics and objects (Zaichkowsky, 1986), for example, divided it into three dimensions based on their objects, advertising involvement, product involvement, and purchase decision making involvement, creating a system that has been widely used. He moreover defined the product involvement as the degree to which the customers are willing to pay for the goods they need. Hong (2015) studied the effects of situational involvement, perceived risk and trust expectation on the consumer's choice of an online merchant. They find that consumer's trust expectation in an online merchant is a predictor of the consumer's choice between an e-tailer and an e-marketplace. Rokonuzzaman et al. (2020) studied the link between consumer's product involvement and store loyalty with many mediating factors like product quality, service quality, and information search. They found that the effect of product involvement on store loyalty is mediated by product quality. In addition, information search works as a mediator between product involvement and store loyalty. Both product quality and information search work as serial mediators between product involvement and store loyalty. Sharma and Klein (2020) investigated the perceived value and involvement in online group buying activities for interpersonal influence. Peng et al. (2019) investigated the role of time pressure and product involvement in the relationship between perceived value and purchase intention. This reveals that perceived value is positively related to purchase intention, whereas time pressure negatively moderates the effect of emotional/social value on purchase intention.

The existence of consumer self-perception is based on the product involvement, therefore, many studies suggest that the impacts for consumers toward products depends on their own interests, values and other characteristics (e.g., Gan and Wang, 2017; Zheng et al., 2017; Rokonuzzaman et al., 2020; Sharma and Klein, 2020). The higher the product involvement level of the consumer, the more important the product is to the consumer. According to the consumers' products involvement levels, the consumers can be divided into low involvement consumers and high involvement consumers. Compared to consumers with low product involvement, the perceived risk of consumers with high product involvement is usually higher. Moreover, consumers with high product involvement will be more eager to look for valuable information, which results in better final purchase decision-making behavior compare to the consumers with low product involvement. Based on the above analysis, we find that product involvement has a certain influence on the perceived value, perceived risk, and purchasing decision of the consumer.

At the end, summarizing, we can say that the perceived value is the most important factor affecting the consumer's decision and that different degrees of involvement lead to differences in consumer perceived value and perceived risks, which ultimately affect the final decision making.

Hence, we propose the following hypothesis 5.

**Hypothesis 5**. *Product involvement has a moderating effect on perceived value, perceived risk, and channel selection*.

## 3. Methodology and Research Design

### 3.1. Ethics Approval

Ethical review and approval was not required for the study on human participants in accordance with the local legislation and institutional requirements.

### 3.2. Consent Statements

Written informed consent from the participants was not required to participate in this study in accordance with the national legislation and the institutional requirements.

### 3.3. Methodology

**Figure 1:**
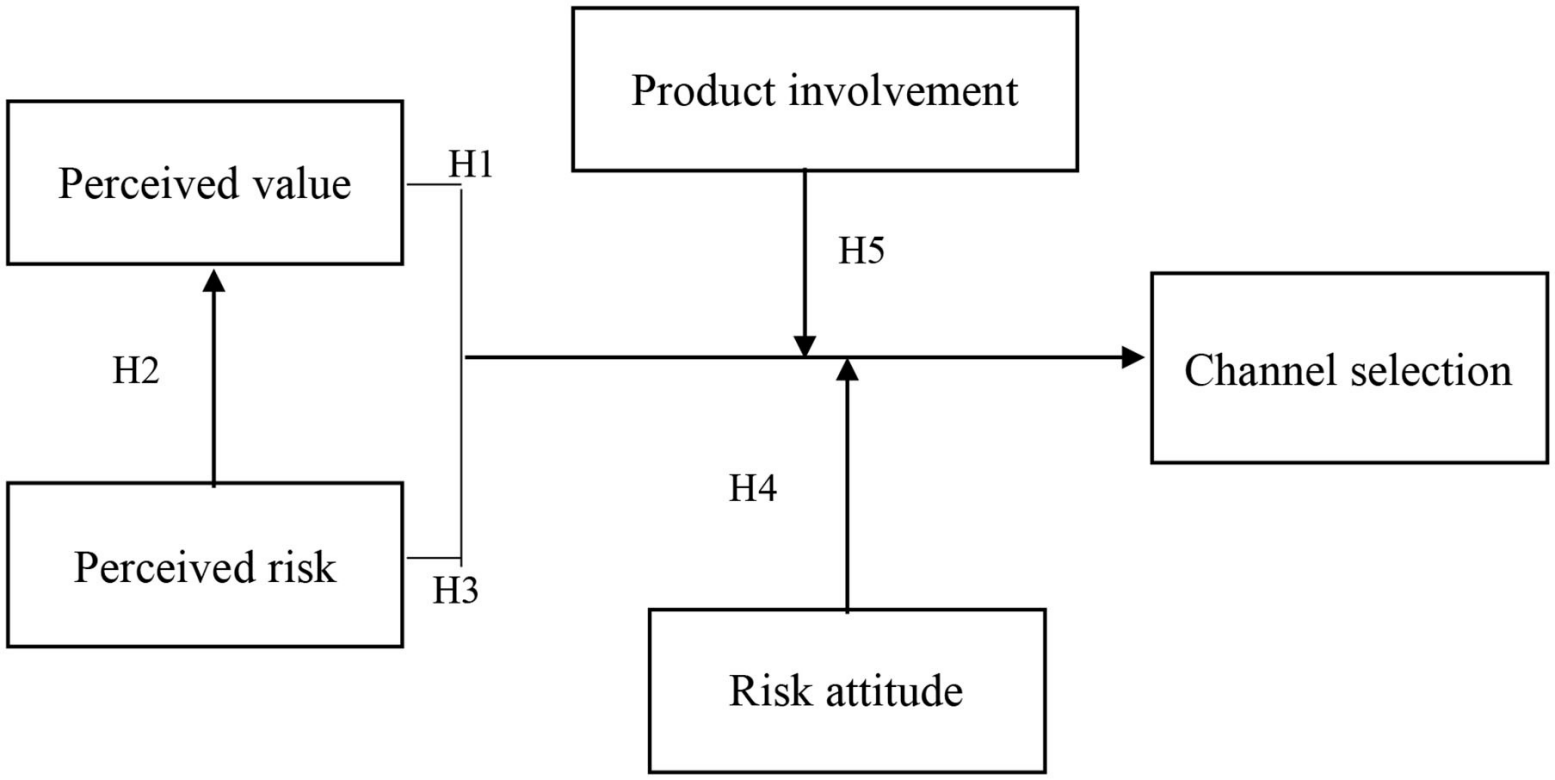
Research hypothesis model.

In this study, we summarized the major research-related theories and formulated the following research framework in Figure 1, consisting of five hypotheses with five variables. Here, we use an adjusted version of the original measurement methodology from Pine and Gilmore (2001) and Featherman and Pavlou (2003) in which they designed the measurement parameters with respect to perceived value, perceived risk, product involvement, risk attitude, and channel selection.

Following, we formulate a final questionnaire, based on the literature review and the initial survey results, divided into three parts. The first part is designed in order to access the consumer's online shopping channel selection for customized garment product (Jo Anderson-Connell et al., 2002; Peña-García et al., 2020). The second part aims to measure the perceived value and perceived risk derived by the retailer's channel selection for customized garment product (Dowling, 1986; Masoud, 2013; Chiu et al., 2014). The third and last part is intended from the collection of the respondents' personal information (Sharma and Klein, 2020). We use the five-point Likert scale system as follows: strongly disagree (1), neutral (3), and strongly agree (5).

### 3.4. Sampling and Data Collection

In this study, we collaborated with a fashion textile and apparel company that aims to implement its customized product lines within the current year.

The data analyzed were collected from online shopping users: 346 questionnaires were randomly distributed among consumers who previously purchased from some popular customized products (C2B, C2M) websites and apps in China (Biyao, Taobao, Hongling, MIID, UDZ, YBRen, etc.). Respondents were selected from experienced customized products buyers, and they were questioned on the social network apps with which they were familiar as well as similar ones. This survey was conducted from January to February 2017, and out of the 346 questionnaires, 158 were usable for our analysis. Our response rate is 45.66% and the respondents were 35.44% male and 64.56% female (see Table 1).

**Table 1:** The descriptive statistics analysis results of sample features.

**Variables**	**Statistical characteristics**	**Frequency**	**Percentage (%)**
Gender	Male	56	35.44
	Female	102	64.56
Age	15–20	15	9.49
	21–25	115	72.78
	26–30	25	15.82
	31–35	2	1.27
	35 or older	1	0.63
Education background	Middle school	2	1.27
	High school	6	3.80
	University	118	74.68
	Post-graduate or more	32	20.25
Online shopping experience	1 year or below	2	1.27
	1–3 years	44	27.85
	3–5 years	63	39.87
	5 years and above	49	31.01
Monthly expense online shopping	200 CNY or below	16	10.13
	200–600	66	41.77
	600–900	38	24.05
	900–1,200	16	10.13
	1,200–1,500	10	6.33
	1,500 or above	12	7.59
Online shopping channel	Official website/app	105	66.46
	Platform	53	33.54

### 3.5. Data Quality Analysis

#### 3.5.1. Reliability Assessment

Whether the data are reliable or valid is the premise of the hypothesis testing. In this paper, SPSS is used to analyze the reliability (using Cronbach's α coefficient) and validity of the data. Prior to the consistency check of the questionnaire, the Corrected Item-Total Correlation (CITC) was used to delete the unqualified items (when the CITC value is <0.5) (Chen et al., 2020). The analysis results show that, except for the index PV6 (0.476) (PV represents Perceived Value), which is used for measuring value and the index PR6 (0.444) (PR represents Perceived Risk) used for measuring risk, the CITC value of the other items are all >0.5. The reliability testing result after removing the unqualified items can be found in Table 2. The α value of each variable was >0.8, which means the variables, after the adjustment, has a higher reliability.

**Table 2:** The reliability testing results of each variables.

**Variables**	**No**.	**CITC**	**CAID**	**Cronbach's α value**
PV	PV1	0.652	0.886	0.898
	PV2	0.608	0.888	
	PV3	0.565	0.890	
	PV4	0.663	0.885	
	PV5	0.637	0.887	
	PV7	0.687	0.884	
	PV8	0.616	0.888	
	PV9	0.647	0.886	
	PV10	0.617	0.888	
	PV11	0.621	0.887	
	PV12	0.586	0.890	
PR	PR1	0.706	0.824	0.860
	PR2	0.678	0.830	
	PR3	0.625	0.839	
	PR4	0.647	0.835	
	PR5	0.561	0.851	
	PR7	0.684	0.828	
CS	CS1	0.635	0.761	0.816
	CS2	0.715	0.686	
	CS3	0.647	0.769	

#### 3.5.2. Validity Evaluation

The evaluation of data validity is mainly divided into content validity and construct validity. The design of variable dimensions and indicators is based on a pre-investigation and has met repeated corrections.

Firstly, we use SPSS for the KMO measure and the Bartlett sphere test to determine whether the sample data obtained from the questionnaire can be taken into count in our factor analysis. Secondly, we use the factor analysis of the variables to test the construct validity. Table 3 is the result of the KMO measure and the Bartlett sphere test. Table 4 shows the results of the principal component analysis. As we can see, the KMO measure value of the sample reaches 0.872, the KMO measure of each variable is above 0.7, the KMO measure of perceived value and perceived risk is 0.85 or more, and the significance level of the χ^2^ statistic of the Bartlett sphere test is 0. These data indicate that the sample is suitable for factor analysis: the most widely used method of verifying construct validity (Field, 2013), of which the principal component analysis is a commonly used factor analysis method.

**Table 3:** The KMO measurement Bartlett test of sphericity and for variables.

**Variables**	**KMO**	**Bartlett test of sphericity**		
		**Chi-squared approximation**	**df**	**Sig**.
PV	0.886	924.362	66	0.000
PR	0.868	450.399	21	0.000
CS	0.706	173.484	3	0.000
Total	0.872	1815.358	231	0.000

**Table 4:** The result of confirmatory factor analysis.

**Variables**	**No**.	**Factor loadings**	**Reliability coefficient *R*^2^**	**Composite reliability**	**AVE**
PV	PV1	0.706	0.499	0.8564	0.51
	PV2	0.647	0.418		
	PV4	0.824	0.679		
	PV5	0.819	0.671		
	PV7	0.640	0.409		
	PV11	0.583	0.340		
PR	PR1	0.832	0.692	0.84	0.57
	PR2	0.818	0.669		
	PR4	0.654	0.428		
	PR7	0.692	0.479		
CS	CS1	0.728	0.530	0.82	0.61
	CS2	0.858	0.736		
	CS3	0.735	0.541		

Through the principal component analysis, it is found that the amount of principal component with the eigenvalue >1 is three, which is consistent with the number of variables set up in this paper, and the total variance of the three principal components is 61.43%, which is 60% higher than the common standard. It can therefore be shown that the construction validity of observed variables is better.

### 3.6. Model Estimation and Evaluation

#### 3.6.1. Confirmatory Factor Analysis

When using the structural equation model to verify the hypotheses, it is necessary to determine the relationship between latent variables. We usually use the confirmatory factor analysis to evaluate the combination reliability (CR) of the latent variable, and then, with the CR, we test whether each observation variable can reflect the latent variables correctly and completely. The reliability coefficient (R2) and the average variance extraction (AVE) of each index are used to measure to what extent these indexes can be explained by the latent variable. The test indicates that the internal quality of the model is sufficient when the R2 value is >0.3 and that the index can reflect the potential traits of common factors validly when the AVE value is >0.5. Moreover, it shows that the internal quality of the model is adequate when the CR value is higher than 0.6 (Peng et al., 2019).

Index PV3, PV8, PV10, PV12, PR3, and PR5 have all been deleted based on the results of confirmatory factor analysis available in Table 4. The CR value of PV, PR, and CS are all >0.8, and the AVE value is >0.5, which can finally verify the quality of the latent variables.

#### 3.6.2. Fitness Evaluation of Causal Relationship Model

In order to test the above-proposed theoretical hypotheses, the intrinsic quality of the latent variable model is first modified and then evaluated with the maximum likelihood estimation method.

**Figure 2:**
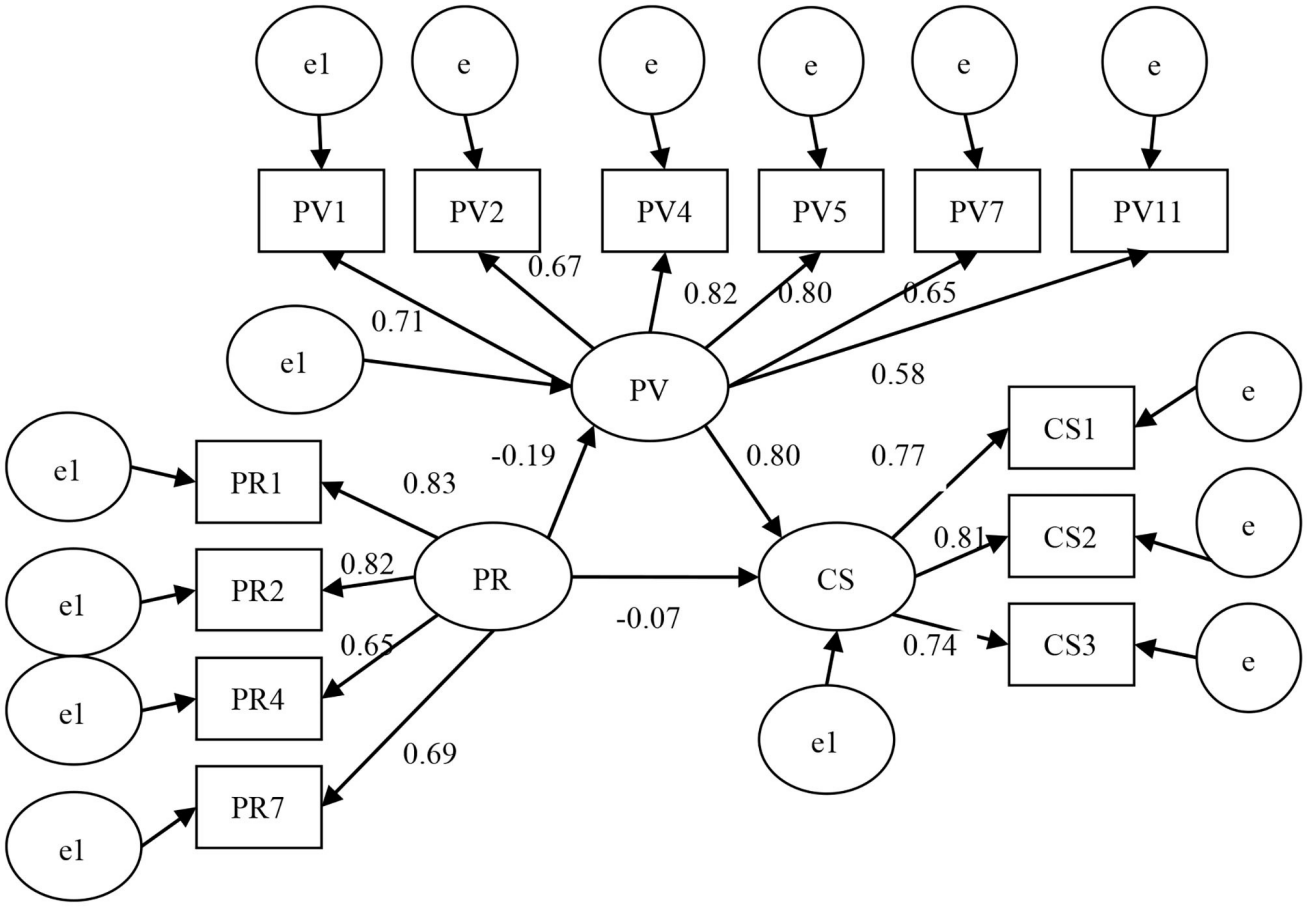
The model with standardized parameters.

After verification, we determine whether the relevant index value meets the conditions. Furthermore, we delete or modify the unreasonable path to achieve the optimization of the hypothetical model. Figure 2 is the revised theoretical model.

In this paper, Table 5 shows the evaluation of the overall model's quality while Table 6 presents the test results of the theoretical hypothesis. As shown in Table 5, except that the CN value (180) is <200, the other quality indicators are all greater than the standard value of 0.9; in detail, the χ^2^ have a significance level of 0.12 > 0.05, the χ^2^ degree of freedom ratio is 1.214 < 2, the RSEMA value is 0.036 < 0.05; the GFI value is 0.935, AGFI value is 0.905, IFI value is 0.986, and TLI value is 0.986. All these values show that the adaption greater than the standard value of 0.9. It shows that the adaptation situation of the whole model has reached the optimal level and that there is a certain matching degree between the questionnaire data and the hypothesis. In Table 6, perceived risk is negatively correlated with both perceived value and channel selection, while, of course, perceived value and channel selection have a significant positive correlation.

**Table 5:** The evaluation of overall goodness of fit.

**Statistical indicator**	**SI of χ^2^ (P)**	**χ^2^/*df***	**CN**	**RSEMA**	**GFI**	**AGFI**	**IFI**	**TLI**	**CFI**
Indicators	0.12	1.214	180	0.036	0.935	0.905	0.986	0.982	0.986

**Table 6:** The hypothesis testing result.

**SEM Path**	**Standardized path coefficient**	**S.E**.	**t value (C.R.)**	**P**	**Result**
PR → PV	−0.187	0.065	−2.097	0.036	Support
PV → CS	0.799	0.132	7.510	^***^	Support
PR → CS	−0.070	0.049	−2.315	0.022	Support

In Table 6 above, * means obvious on 0.05 level, ** means obvious on 0.01 level, *** means obvious on 0.001 level.

#### 3.6.3. Regulation Test

In order to examine the regulating effect of product involvement on channel selection, in this paper, we firstly divide the product involvement of the research object into high and low degrees of involvement. The overall mean value of the product involvement, which is calculated from four items is 3.91, and the standard deviation is 0.67.

The overall data are then divided, on the base of the mean value, into two groups: the low involvement group, which includes 71 sets of data, and the high involvement one, which includes 96 sets. Following, we carried out a single factor analysis based on the variance which showed that there was a significant difference between the two groups, and an AMOS analysis, which was used to examine the regulating effect after the grouping process.

The results of both unrestricted and restricted models were then compared with the all group analysis. We can see in Table 7 that the χ^2^ value of test was significant (0.033 < 0.05), which means we can reject the null hypothesis and accept the opposite hypothesis: the product involvement has a significant regulating effect.

**Table 7:** The comparison result of default model and restricted model with respect to high involvement and low involvement.

**Model**	**DF**	**CMIN**	**P**	**NFI δ_1_**	**NFI δ_2_**	**RFI ρ_1_**	**RFI ρ_2_**
Moderating role	3	8.705	0.033	0.009	0.011	0.005	0.006

Similarly, the χ^2^ value of test in Table 8 was not significant (0.265 > 0.05), which means the regulating effect of risk attitude to consumer channel choice is not meaningful.

**Table 8:** The comparison result of default model and restricted model with respect to risk aversion and risk preference.

**Model**	**DF**	**CMIN**	**P**	**NFI δ_1_**	**NFI δ_2_**	**RFI ρ_1_**	**RFI ρ_2_**
Moderating role	3	3.970	0.265	0.004	0.004	0.000	0.000

## 4. Conclusions and Research Implications

Given the data collected from the questionnaires, the following conclusions are achieved with respect to the analytical results from AMOS:

Conclusion 1: Based on the descriptive statistics, nowadays, consumers, when choosing customized fashion garment online shopping channels, have a preference for those official direct ones. Presumably, the phenomenon is due to the fact that the majority of the consumers tend to believe that it is less risky to use an official direct channel for customized products. Moreover, this kind of online shopping pattern conforms the most to the traditional concept of purchasing.

Conclusion 2: Consumers' perceived value of online shopping channel has a positive impact on their choice making. The data analysis shows a relatively strong positive correlation between perceived value and channel selection, which means that when a consumer is selecting online shopping channels for customized fashion garments, according to people's usual logical thinking, then almost all the functional, sentimental, and social values are affecting their consideration. Furthermore, the perceived values of direct channels outrange the ones people perceive from the third-party retail channels universally and, thus, help consumers in making their final purchase decisions.

Conclusion 3: Consumers' perceived risk and shopping channel involvement in customized fashion garment have both a negative impact on the perceived value and the channel selection. Statistics, together with many other research findings, indicate that both perceived value and channel selection procedure correlate negatively with perceived risk to a large extent. However, the data presented in this study show a rather poor negative correlation coefficient, which may be the result of the online channel customized fashion garment platforms' policy taken to increase the coefficient of safety and to protect consumers' rights. Additionally, the findings show that among most consumers' opinions, those official direct channels for customized products are less risky than their traditional counterparts.

Conclusion 4: Product involvement has a moderating effect on the relationship between channel's perceived risk, perceived value, and channel selection process; this shows how fundamental the customized product is to the consumer and how much attention the consumer pays to the customized product influence on consumers' perception. All of this helps to regulate the relationship mentioned above and eventually might have an influence on the channel selection and purchase decision process for the customized products.

Based on the related literature of online shopping and channel selection of customized garment products, this paper penetrates the matter focused on how consumers choose their online shopping channels from the perspective of perceived values, constructs a theoretical model composed of perceived values, perceived risk, and channel selection, and brings forth new ideas in the moderating effects of risk attitude and product involvement.

The analysis of the sample data with the use of AMOS and SPSS proves the influence posed by both perceived value and risk, as well as product involvement, on the multi-channel online shopping environment. Besides, the research results show that although perceived value and perceived risk affect the choosing procedure, there are still some differences between the new online multi-channel environment and the regular one.

Most studies concerning channel choices focus mainly on offline, traditional and online parts while this paper provides an in-depth understanding of the choosing procedures between different online channels and take consumer's personal factors for customized garment products into consideration, which means it fills the existing gap in the problem of online shopping channel selection, offering new ideas for the development of online multi-channel environments. Similarly, the rapid advancement of online shopping for customized garment products will have an impact on the sellers frustrated about choosing the most beneficial channel to sell their customized products.

Given the findings, there is no doubt that the key to soaring online shopping sales for customized garment products remains on consumers. And, thus, online sellers ought to further clarify the features and positioning of their customized products due to the impact of several factors like product involvement on consumers' decision-making process and to realize the features and positioning of the channels. They must focus on the importance of perceived value and risk to make optimal use of the channels for customized garment products by lowering the risk and improving the revenue.

As for the various online shopping channels for customized garment products, a high-quality, raffinate, cost-effective product can indeed attract consumers and maintain sustainability in the industry.

In future studies, we can extend this issue by increasing sample size to re-study and re-verify the related factors. In addition, another future studies path will focus on whether or how purchase decisions for customized garment products are influenced by consumers' strategic behavior, internal mental status, and risk attitude.

## Data Availability Statement

The raw data supporting the conclusions of this article will be made available by the authors, without undue reservation.

## Ethics Statement

Ethical review and approval was not required for the study on human participants in accordance with the local legislation and institutional requirements. Written informed consent from the participants was not required to participate in this study in accordance with the national legislation and the institutional requirements.

## Author Contributions

BD: conceptualization. JH: investigation. ZL, BD, JY, and WY: writing of the original draft preparation. BD and LP: writing, review, and editing. BY: methodology. YZ: funding acquisition. All authors have read and approved the final manuscript.

## Conflict of Interest

The authors declare that the research was conducted in the absence of any commercial or financial relationships that could be construed as a potential conflict of interest.
